# The Properties and Domain Requirements for Phase Separation of the Sup35 Prion Protein In Vivo

**DOI:** 10.3390/biom13091370

**Published:** 2023-09-10

**Authors:** Bryan Grimes, Walter Jacob, Amanda R. Liberman, Nathan Kim, Xiaohong Zhao, Daniel C. Masison, Lois E. Greene

**Affiliations:** 1 Laboratory of Cell Biology, National Heart, Lung and Blood Institute, National Institutes of Health, Bethesda, MD 20892, USA; 2 Laboratory of Biochemistry and Genetics, National Institute of Diabetes and Digestive and Kidney Diseases, National Institutes of Health, Bethesda, MD 20892, USA

**Keywords:** phase separation, biomolecular condensates, yeast prion protein, Sup35, prion domain

## Abstract

The Sup35 prion protein of budding yeast has been reported to undergo phase separation to form liquid droplets both at low pH in vitro and when energy depletion decreases the intracellular pH in vivo. It also has been shown using purified proteins that this phase separation is driven by the prion domain of Sup35 and does not re-quire its C-terminal domain. In contrast, we now find that a Sup35 fragment consisting of only the N-terminal prion domain and the M-domain does not phase separate in vivo; this phase separation of Sup35 requires the C-terminal domain, which binds Sup45 to form the translation termination complex. The phase-separated Sup35 not only colocalizes with Sup45 but also with Pub1, a stress granule marker protein. In addition, like stress granules, phase separation of Sup35 appears to require mRNA since cycloheximide treatment, which inhibits mRNA release from ribosomes, prevents phase separation of Sup35. Finally, unlike Sup35 in vitro, Sup35 condensates do not disassemble in vivo when the intracellular pH is increased. These results suggest that, in energy-depleted cells, Sup35 forms supramolecular assemblies that differ from the Sup35 liquid droplets that form in vitro.

## 1. Introduction

Yeast have about a dozen prion proteins, which are characterized by having a properly folded conformation and an infectious prion or amyloid conformation. In addition to the prion proteins identified in yeast, proteomics studies have shown that there are more than a hundred proteins with prion-like domains in yeast [1]. Prion-like domains share a similar amino acid composition to the prion domains of Sup35, Ure2, and Rnq1, which form the [*PSI*^+^], [*URE3*], and [*PIN*^+^] prions, respectively. In the cell, prion-like domains function in protein–protein interactions that drive the phase separation of proteins. When phase separation occurs in a cellular environment, it produces biomolecular condensates. Biomolecular condensates provide a mechanism for compartmentalizing and concentrating functionally related components in membrane-less organelles such as centrioles, but they have also been shown to cause many neurodegenerative diseases [2]. In vitro, phase separation occurs when proteins reach a critical concentration, and they form viscous liquid droplets.

Recognizing the importance of prion-like domains in phase separation and condensate formation, the Alberti laboratory examined whether the prion domain is like the prion-like domain in driving the phase separation of proteins into liquid droplets [3]. To test this, they used the canonical yeast prion protein Sup35. Sup35 is an essential multidomain protein that functions to terminate translation of mRNA. The N-terminal region of Sup35 is a prion domain, which is an intrinsically disordered domain enriched in glutamine and asparagine residues, folds into an in-register parallel beta-sheet when Sup35 forms an infectious prion [4], while the essential C-terminal domain of Sup35 binds to Sup45 to make the translation termination complex [5,6,7]. These domains are linked by the highly charged M- or middle domain, which has been shown to bind Hsp104 and affect the stability of the amyloid fibers [8]. When the N-terminal domain of Sup35 misfolds into the infectious amyloid conformation, Sup35 forms the [*PSI*^+^] prion, which markedly reduces the efficiency of translation termination in yeast [9,10,11,12,13,14].

The Alberti laboratory showed that purified Sup35 underwent a reversible phase separation causing the formation of liquid droplets in vitro [3]. Furthermore, domain analysis showed that both the NM domains and MC domains of Sup35 formed liquid droplets at low pH, but the MC domains formed droplets to a lesser extent than the full-length Sup35. However, the C-terminal domain alone irreversibly aggregated at low pH. By mutating the highly charged M-domain of Sup35, they also found that this domain regulated the pH dependence of Sup35 phase separation. Based on these in vitro observations, they proposed that the N-terminal domain of Sup35 provides the driving force for liquid droplet formation and the M-domain acts as a stress sensor [3].

Having established that Sup35 phase separates into liquid droplets in vitro, they examined whether Sup35 also phase separates into liquid droplets or condensates in vivo by imaging fluorescently labeled Sup35 when the cytosol was acidified [3]. Consistent with their in vitro results, when the yeast were subjected to either pH stress or energy depletion, both methods that decreased the intracellular pH, the Sup35 formed condensates. Furthermore, the condensates were disassembled upon glucose addition, which, by producing ATP, raised the intracellular pH. Similar to their in vitro results, they showed that it is the M-domain of Sup35 that regulates the pH dependence of Sup35 condensate formation in the yeast. When only the C-terminal domain was expressed in yeast, it formed fluorescent puncta at low intracellular pH, but these puncta disassembled at a significantly slower rate upon addition of glucose than the Sup35 condensates. This suggested that, as occurred in vitro, the C-terminal domain formed irreversible aggregates at low intracellular pH. They also found that, following acidification of the cytosol, the growth recovery of the yeast was faster in yeast expressing full-length Sup35 than in yeast expressing only the C-terminal domain of Sup35. Based on these observations, the Alberti laboratory proposed that the physiological function of the Sup35 condensates in vivo is to promote cellular fitness [3,15].

Not only does energy depletion and low pH cause Sup35 condensate formation, but these conditions promote other proteins to form condensates such as stress granules and P-bodies, [16,17]. Stress granules and P-bodies are composed of many proteins with intrinsically disordered domains, RNA binding proteins, and RNA itself [16,17,18,19,20,21,22]. In fact, analysis of yeast stress granules by mass spectroscopy revealed that they contain several hundred proteins [23,24,25]. Sup35 and Sup45 were found in stress granules that formed after heat shock [24,25,26], but not in stress granules formed by stressing yeast with sodium azide [23]. Sup35 and Sup45 were also detected in P-bodies; in contrast to stress granules, P-bodies, which are thought to be precursors of stress granules [16], are present in non-stressed cells, but become larger when the cells are stressed, e.g., by glucose starvation [27,28]. Additionally, Sup35 condensates partially colocalized with Pab1, another protein marker for stress granules [16], in yeast with low intracellular pH [3]. Therefore, even though the N-terminal prion domain of Sup35 has an intrinsically disordered conformation that drives it to form liquid droplets in vitro, it is not clear how well the properties of the Sup35 liquid droplets in vitro reflect those of the Sup35 condensates that form in vivo when yeast are energy-depleted.

In this study, we further characterized the Sup35 condensates formed by acidification of the yeast cytosol by energy depletion to determine whether the in vivo condensates are analogous to the in vitro liquid droplets of purified Sup35. In agreement with the Alberti laboratory [3], we found that the full-length Sup35 formed condensates in vivo, but in contrast to what they observed in vitro, the NM domains of Sup35 did not phase separate into condensates when the intracellular pH was acidified. Therefore, the C-terminal domain of Sup35 is required for Sup35 to phase separate into condensates when yeast are energy-depleted. Furthermore, Sup35 condensate formation in vivo apparently requires mRNA, since treating the yeast with cycloheximide, which prevents release of mRNA from the polysomes [29,30,31,32,33], inhibited condensate formation. In addition to RNA, Sup35 condensates in vivo colocalized with Sup45 and Pub1, a stress granule marker protein. These results show that the liquid droplets of Sup35 that form in vitro differ from the Sup35 condensates that form in vivo in energy-depleted cells. In fact, the Sup35 condensates that form in energy-depleted cells appear to be supramolecular assemblies resembling stress granules.

## 2. Materials and Methods

### 2.1. Yeast Strains and Plasmids

Yeasts were grown at 30 °C on synthetic-defined medium (SD, 0.7% yeast nitrogen base, 2% glucose) with complete supplement mixture or the appropriate amino acid dropout for selection and maintenance of the plasmid (Sunrise Scientific, Knoxville, TN, USA). The yeast strains used in this study listed in Table 1 were all cured of the [*PSI*^+^] prion, which we verified by plating the yeast on ½ YPD plates. On these plates, [*PSI*^+^] is detected due to mutations in either the *ade1* or *ade2* gene in our yeast strains, which gives rise to red colonies when yeast are [*PSI*^+^] and white colonies when the yeast are cured. The plasmids used in this study are listed in Table 2.

The 1618 C-GFP strain was obtained by transforming the 780-1D strain, which expresses Sup35 on a on a *URA3* plasmid with pAG415-P*_SUP35_*-*SUP35-C-GFP*. Cells were grown in medium with 5′-fluoro-orotic acid (U.S. Biological, Salem, MA, USA) to shuffle out the URA3 plasmid. To engineer strain 1905, which has Sup45-GFP integrated into the *SUP45* locus of strain 779-6A, we used the GFP–yeast clone YBR143C (Thermo Fisher Scientific, Waltham, MA, USA) to clone the *SUP45*-*GFP-HIS3MX6* module using the following primers: forward primer (−516 to −489) GCTGGATATGAGCAGTATGAGGTAGACC and the reverse primer (+331 to +354) GACAGGTGGGCTAGTGAATTTAGC. This module was then integrated into the 779-6A yeast strain in the *SUP45* chromosomal locus. The integrated allele was verified by sequencing PCR products amplified from candidate transformants. Verified candidates were backcrossed.

The plasmid p415-P*_SUP35_*-*SUP35-NM-GFP* was subcloned from pAG415-P*_SUP35_-SUP35-GFP* [3] by deleting amino acids 253–685 from the full-length protein. This was accomplished by fusing the NM fragment with the BamH1 linker on the C-terminal GFP using the following forward primer: GAAGTGGATGACGAAGTTGTTAACGGATCCGCTGGCTCCGCTGCTG.

### 2.2. Conditions for Condensate Formation

Yeast cells were grown overnight to an OD 0.45–0.60. They were centrifuged, washed in PBS, and then energy-depleted for 90 min as described previously [3]. Cells were energy-depleted in SD medium without glucose containing 20 mM 2-deoxy-D-glucose (Sigma, St. Louis, MO, USA) and 10 µM Antimycin A (Sigma). When cells were energy-depleted at neutral pH, we used the same energy-depletion medium with buffer added to raise the pH. We routinely added 1 M imidazole, pH 7.5 to the medium to obtain a final concentration of 50 mM imidazole. In some experiments, cells were incubated in the energy-depletion medium for 90 min, followed by addition of buffer to neutralize the medium. In experiments with cycloheximide, 100 µg/mL of cycloheximide (Sigma) was included in the PBS wash. In several experiments, the intracellular pH of yeast was acidified by resuspending the yeast after the PBS wash into 2 mM, dinitrophenol (DNP) and 100 mM phosphate (pH 5.0), as described previously [3].

### 2.3. Recovery from Energy Depletion

After incubating the yeast in energy-depletion medium or DNP/phosphate buffer (5.0) for 90 min, the cells were spun down and resuspended in SD full medium with glucose at an OD_600_ of ~0.1. The cells were added to 96-well tissue culture plates (Sarstedt, Nümbrecht, Germany) and then placed into the Synergy H1 microplate reader (Biotek, Waltham, MA, USA). The absorbance was measured at 10 min intervals for 24 h. The plates were maintained with constant shaking at 30 °C.

### 2.4. Intracellular pH Measurements

Intracellular pH was measured by transforming yeast with the sfpHluorin plasmid [34]. Cells were grown in methionine-free medium to induce expression. Prior to measuring the intracellular pH of the yeast, we made a standard curve using buffers ranging from pH 5 to pH 8 using 2 M Na_2_HPO_4_ and 0.1 M citric acid solutions. These different pH standards were added to the yeast, which were treated with digitonin to permeabilize the membrane [35].

Fluorescence excitation spectra were recorded with a fluorescence spectrometer (LS55, Perkin Elmer, Waltham, MA, USA). The excitation spectra for the different pH values were recorded in 1 mL quartz cuvettes at room temperature using the LS55 fluorescence spectrometer (settings: emission wavelength 508 nm, excitation wavelength 350 to 490 nm).

### 2.5. Confocal Microscopy

Fluorescence images were obtained on the Zeiss LSM 880 Airyscan confocal microscope equipped with a 63x/1.40 oil objective. Z-stack confocal images were taken of all cells. The yeast were routinely imaged in 8-well 25 mm^2^ chambered coverslips (Cellvis, Mountain View, CA, USA). Photobleaching of the GFP fluorophore was accomplished by bleaching the same region of interest 10 times with the 488 nm laser at 100% for a total bleach time of 1 s. After bleaching, images were acquired every 5 s. The change in fluorescence intensity of the bleached and non-bleached regions were quantified using the Zeiss Zen 3.3 program. The initial fluorescence intensities of the bleached and non-bleached areas were set to 1 to normalize the data.

Colocalization of proteins were calculated using the Imaris 9.2 program (Bitplane, South Windsor, CT, USA), in which we which first identified red and green spots, followed by using the colocalization program to determine the extent of overlap between the red and green spots. The Supplement lists the Imaris parameters (Table S1) used in identifying red and green spots and in measuring the colocalization between red and green spots. The program was used to analyze colocalization in a minimum of 250 cells for each experimental condition.

## 3. Results

### 3.1. Energy Depletion of Yeast Expressing Either GFP-Labeled Sup35 or NM-GFP

It was previously shown by the Alberti laboratory that the properly folded conformation of Sup35 phase separates at acidic pH both in vitro and in vivo [3]. Based on their study of the domains of Sup35, they concluded that this phase separation was caused by the prion domain of Sup35 and was regulated by the highly charged pH-sensitive M-domain. To further our understanding of the phase separation of yeast prion proteins, we extended the characterization of the Sup35 condensates in vivo, which they demonstrated were formed by decreasing the intracellular pH of the yeast cytosol. In their study, they used two different methods to reduce the pH, both of which yielded the same results. They either added 2 mM DNP in 100 mM phosphate buffer (pH 5.0) or SD medium without glucose containing 20 mM deoxyglucose and 10 µM antimycin A. We routinely used the latter method in our experiments, referred to here as energy depletion, to reduce the intracellular pH, because DNP has the secondary effect of depolarizing membranes [36], which disorganizes the lipid microdomains on the plasma membrane [37].

**Figure 1:**
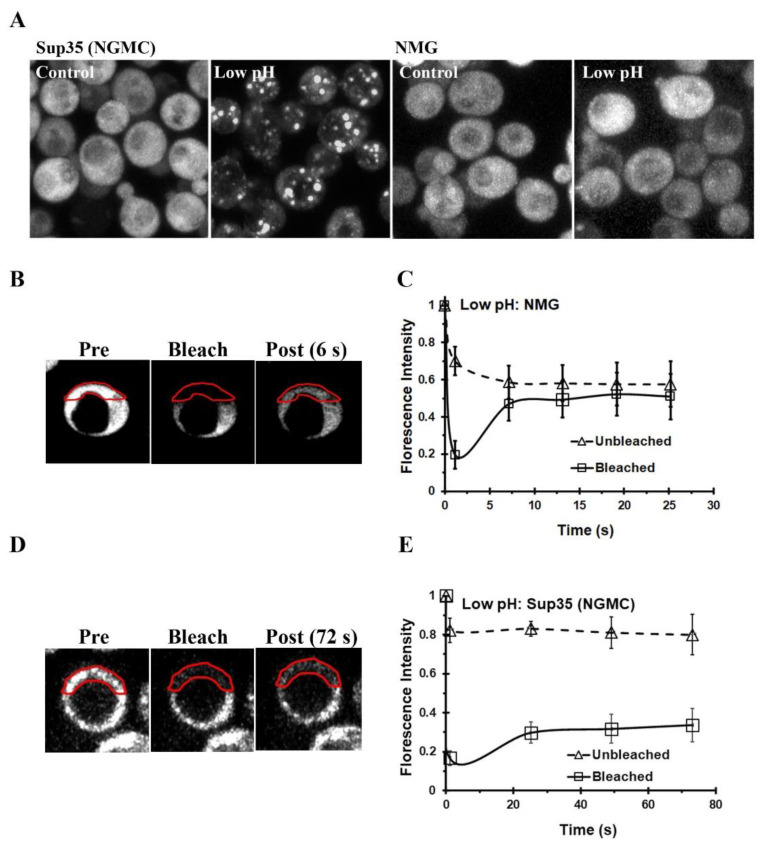
Acidification of the intracellular cytosol by energy depletion causes phase separation of Sup35, but not the NM domains of Sup35. (**A**) Maximized Z-stack confocal images of yeast expressing either Sup35 (NGMC) or NM-GFP (NMG) were taken before energy depletion and 75 min after energy depletion. (**B**) Images of energy-depleted yeast expressing NMG before photobleaching, directly after photobleaching, and following photobleaching of energy-depleted yeast. The red line indicates the bleached region in the yeast cell. (**C**) Plot of the change in fluorescence intensity of NMG following photobleaching of energy-depleted yeast. The intensity was measured both in bleached and unbleached regions as a function of time. The data were normalized to obtain an average and standard deviation (n = 5). (**D**) Images of energy-depleted yeast expressing Sup35 (NGMC) before photobleaching, directly after photobleaching, and 72 s following photobleaching. (**E**) Plot of the change in fluorescence intensity of NGMC following photobleaching of energy-depleted yeast. The intensity was measured both in bleached and unbleached regions as a function of time. The data were normalized to obtain an average and standard deviation (n = 5).

Sup35 condensates were previously identified in yeast by imaging GFP-labeled Sup35 [3], using a yeast strain expressing GFP-labeled Sup35 (NGMC) from the *SUP35* promoter that was engineered in our laboratory. As expected, we also obtained Sup35 condensates when this yeast strain was energy-depleted, which lowers the intracellular pH (Figure 1A). Since a Sup35 fragment consisting of just the N-terminal prion domain and the highly charged M-domain was shown to form liquid droplets in vitro at low pH [3], we expected that the NM fragment would likewise form condensates in vivo when the yeast cytosol was acidified. To test this point, we energy-depleted yeast that contained a plasmid which expressed NM-GFP from the *SUP35* promoter. Measurement of the GFP fluorescence intensity of NM-GFP and NGMC in yeast showed their expression levels were not significantly different. Surprisingly, the NM-GFP had a diffuse appearance following energy depletion, just as it did before energy depletion (Figure 1A). We also observed a diffuse appearance of the NM-GFP when the cytosol was acidified by addition of 2 mM 2,4-DNP and 100 mM phosphate buffer, pH 5.0 (Figure S1A). In addition, when NM-GFP was expressed in a different yeast strain, 74D-694, we obtained similar results. The NM fragment had a diffuse appearance in the energy-depleted yeast, whereas the full-length Sup35 formed condensates under this condition (Figure S1B). Therefore, we did not detect phase separation of NM-GFP in two different yeast strains following energy depletion to acidify the intracellular pH.

Since the NM-GFP appeared diffuse, we expected that this fragment would be mobile in the cytosol, which was tested by photobleaching half of the cell. As shown by the images in Figure 1B, directly following photobleaching, there was a marked decrease in the fluorescence intensity in the bleached region, which recovered in intensity as fluorophores from the unbleached area diffused into the bleached area. When the fluorescence intensity of both the bleached and unbleached regions were plotted as a function of time in Figure 1C, the graph showed that within 6 s after photobleaching the NM-GFP had diffused from the unbleached region into the bleached region. Therefore, the NM-GFP was very mobile in the acidified cytosol when yeast were energy-depleted. In fact, the mobility of NM-GFP was similar to its mobility when cells were incubated in SD medium with glucose (Figure S1C).

In contrast to NM-GFP, the Sup35 condensates were immobile, as shown previously [3]. When we photobleached the full-length Sup35 (NGMC) condensates that form in energy-depleted yeast, there was no fluorescence recovery of the Sup35 condensates after photobleaching (Figure 1D). The lack of fluorescence recovery of the condensates was quantified in Figure 1E. As expected, photobleaching of Sup35 in yeast incubated in SD medium with glucose showed a rapid time course of fluorescence recovery (Figure S1D). Therefore, the NM fragment does not phase separate in vivo, unlike the full length Sup35, composed of NGMC, which indicates that the C-terminal domain of Sup35 is integral for condensate formation in vivo.

### 3.2. Composition of Sup35 Condensates

Since the C-terminal domain of Sup35 is essential for the formation of Sup35 condensates, these results suggest that the C-terminal domain rather than the prion domain of Sup35 is providing the driving force for condensate formation in vivo. One way that this could occur is if the C-terminal domain somehow links to the mRNA since RNA has been shown to be a central hub in the formation of many different condensates including P-bodies and stress granules [16,38,39].

**Figure 2:**
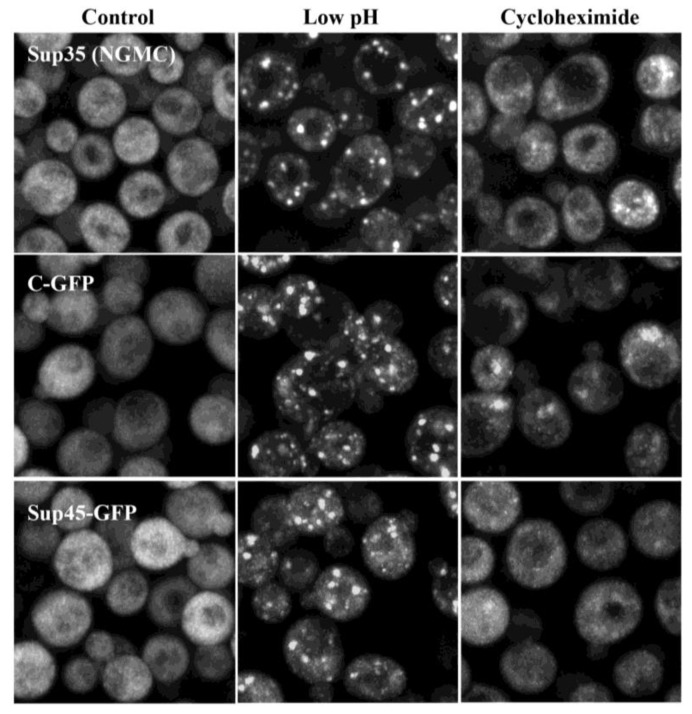
Cycloheximide prevents the formation of Sup35(NGMC) condensates, C-GFP puncta, and Sup45-GFP puncta. Maximized Z-stack confocal images are of cells expressing Sup35 (NGMC), the C-terminal domain of Sup35 (C-GFP), or Sup45-GFP. Cells were imaged in SD full medium or in energy-depletion medium for 75 min, or first treated with cycloheximide followed by energy-depletion medium for 75 min.

To examine whether RNA has such a role in Sup35 condensate formation, the yeast were first treated with cycloheximide to trap the mRNA on ribosomes [15,16,21,40,41,42,43,44], followed by energy depletion of the yeast. As shown in Figure 2, cycloheximide prevented Sup35 from forming condensates. We also examined the effect of energy depletion using a *SUP35*-deletion strain that expressed the GFP-labeled C-terminal domain of Sup35 by itself (C-GFP). When yeast were energy-depleted, Figure 2 shows that the C-GFP formed bright puncta, as was shown previously [3]. However, these puncta did not form when the yeast were treated with cycloheximide, which suggests that both the C-terminal domain of Sup35 and mRNA are important components of Sup35 condensate formation in vivo.

We next examined how the C-terminal domain of Sup35 is linked to the mRNA. One possibility is that the mRNA is linked by Sup45, which complexes with Sup35 to cause translation termination in the yeast [5,6,7]. Sup45 is known to bind to the C-terminal domain of Sup35 [6] and, when yeast are stressed, Sup45 binds to mRNA in a complex with many other proteins [6,11,16,38]. To examine the effect of energy depletion on Sup45, we used yeast that express GFP-labeled Sup45 (Sup45-GFP) from the *SUP45* promoter. As shown in Figure 2, the Sup45-GFP formed bright puncta in energy-depleted yeast, but not when the yeast were treated with cycloheximide prior to energy depletion. Therefore, in order for the Sup45 to form condensates, mRNA must be available, as shown previously [16].

**Figure 3:**
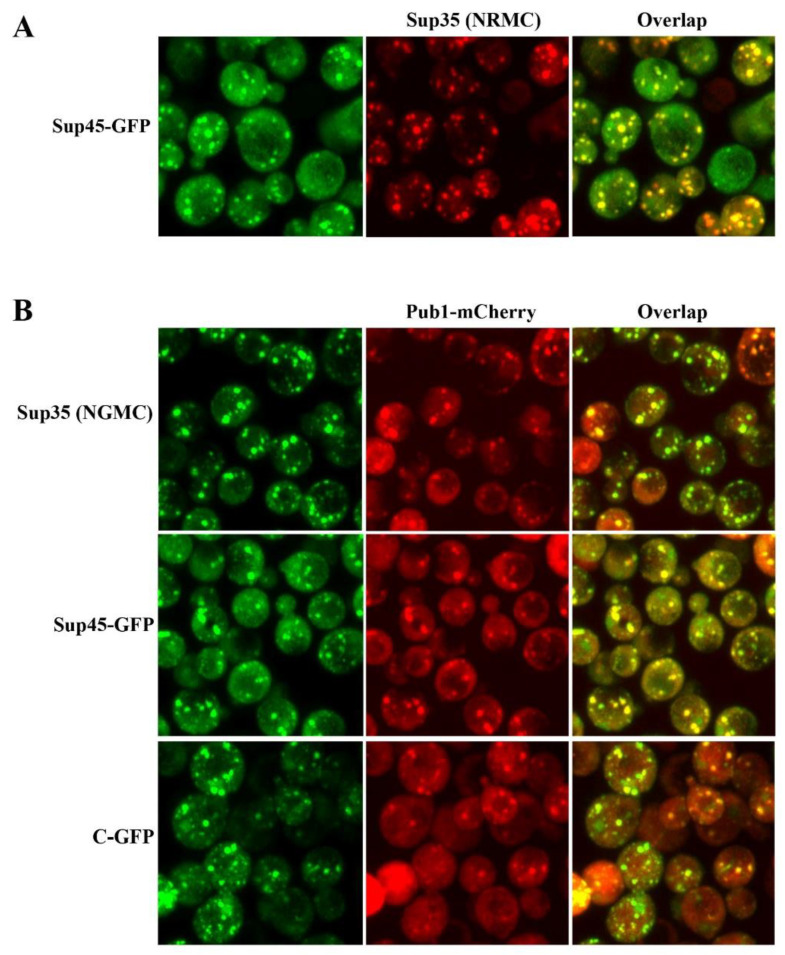
Sup35 colocalized with Sup45 and Pub1 colocalizes with Sup35, C-GFP, and Sup45 when the intracellular pH of yeast was lowered by energy depletion. (**A**) Colocalization of Sup35 with Sup45 imaged in cells expressing Sup45-GFP (strain 1905), which was transformed with a plasmid to express Sup35-RFP (NRMC) in energy-depleted cells. (**B**) Localization of Pub1-mCherry with Sup35 (NGMC), C-GFP, or Sup45-GFP in cells transformed with a plasmid to express Pub1-mCherry. The following cells were transformed with Pub1-mCherry: yeast strain 1074, which expresses Sup35-GFP (NGMC); yeast strain 1618, which expresses C-terminal domain of Sup35 (C-GFP); and yeast strain 1905, which expresses Sup45-GFP.

Since Sup35 binds to Sup45 to make the translation termination complex, we next examined whether the Sup45 colocalized with the Sup35 condensates. This was accomplished by expressing RFP-labeled Sup35 (NRMC) from the *SUP35* promoter in cells expressing Sup45-GFP. Dual color imaging showed extensive colocalization of the Sup35 condensates with the Sup45 puncta (Figure 3A). We found that 72 ± 8% of the Sup35 condensates colocalized with the Sup45 puncta when the images were analyzed using the Imaris program. These results show that when the intracellular pH of yeast was acidified by energy depletion, the Sup35 condensates and Sup45 puncta complexed together, with the C-terminal domain of Sup35 binding to Sup45.

We next examined whether the Sup35 condensates colocalized with Pub1, a stress granule marker protein [16]. It was previously observed that Sup35 condensates partially colocalized with Pab1 [3], which, like Pub1, is used as stress granule marker protein [16]. As shown in Figure 3B, when yeast were energy-depleted, the Sup35 condensates colocalized with the Pub1 puncta in yeast expressing Sup35-GFP and Pub1-mCherry. Similar results were obtained when we energy-depleted cells expressing both Sup45-GFP and Pub1-mCherry (Figure 3B). Data analysis showed that 72 ± 10% of Sup35 foci contained Pub1-mCherry and 76 ± 8% of Sup45 foci contained Pub1-mCherry foci in energy-depleted cells. These colocalization data indicate that Sup35 condensates assemble with Sup45 and Pub1 in a complex with mRNA. We also observed that the C-GFP fragment of Sup35 colocalized with Pub1 (Figure 3B), but to a lesser extent than Sup35 (46 ± 10%). This suggests that the C-terminal domain is like, but not equivalent to the full-length Sup35 in forming these complex assemblies; phase separation of the C-terminal domain is modified by the NM domains of Sup35.

The colocalization of Sup35 condensates with Pub1 suggests that Sup35 is in stress granules. The Parker laboratory reported that stress granules formed within 10 min in glucose-free medium [16], whereas we noticed that in many cells it took about 30 min for Sup35 condensates to form in glucose-free medium (no deoxyglucose or antimycin A). To determine whether stress granules formed at a faster rate than Sup35 condensates in the 779-6A strain, we transformed this strain to express Pub1-mCherry, a stress granule marker protein. Yeast expressing either Pub1-mCherry or Sup35 (NGMC) were incubated in glucose-free medium and imaged at different times. After 15 min in glucose-free medium, essentially all the yeast had Pub1 condensates, whereas only about half of the yeast had Sup35 condensates (Figure S2). With increasing incubation time in glucose-free medium, there was an increase in both the fraction of cells with Sup35 condensates and the number of Sup35 condensates per cell. Therefore, stress granules are formed at a faster rate than Sup35 condensates when yeast are energy-depleted even though, like stress granules, Sup35 condensates contain Pub1 and mRNA.

### 3.3. Disassembly of Sup35 Condensates

Based on our characterization of the Sup35 condensates in energy-depleted yeast, the in vivo condensates are part of a ribonucleoprotein complex composed of many different proteins and mRNA and therefore are very different from Sup35 liquid droplets formed in vitro. We next examined whether increasing the intracellular pH disassembles the Sup35 condensates, which would be expected since there is reversible disassembly of Sup35 liquid droplets with pH in vitro [3]. Addition of glucose to energy-depleted yeast was previously shown to disassemble Sup35 condensates, but it was not examined whether raising intracellular pH in the absence of ATP production is sufficient to disassemble the condensates.

To examine whether raising the pH by itself disassembled the Sup35 condensates in vivo, we first formed the condensates in acidic medium, then added buffer to increase the intracellular pH to neutrality using different buffers to ensure that the buffer itself had no effect on condensate formation or dissolution. The pH was raised to neutrality by adding either 1M imidazole (pH 7.5), 1 M Tris (pH 7.8), or 1 M phosphate buffer (pH 7.5) to a final buffer concentration of 50 mM. Table 3 gives the measured values of the intracellular pH obtained using sfpHlourin [34]. Prior to energy depletion, the yeast had an intracellular pH of ~7.4. After energy depletion in glucose-free medium, the intracellular pH decreased to ~5.4, and this decrease occurred within 20 min. When cells were energy-depleted in glucose-free medium with buffer, which was added either during energy depletion or after energy depletion, the intracellular pH ranged from 6.8–7.4, depending on the buffer.

**Figure 4:**
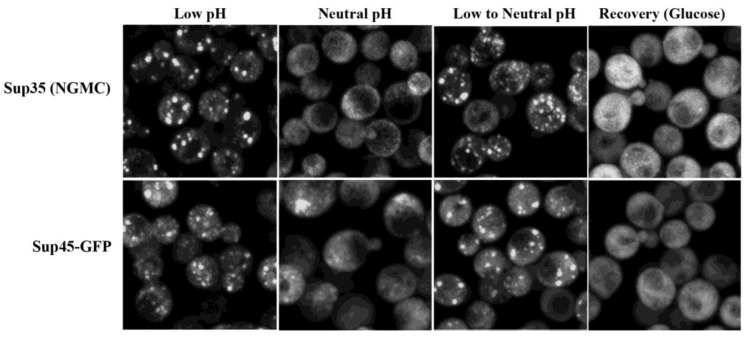
Raising intracellular pH does not disassemble the Sup35 condensates and Sup45 puncta. Maximized Z-stack confocal images are of yeast expressing either Sup35 (NGMC) or Sup45 (Sup45-GFP) under different conditions. In the low pH condition, cells were incubated in energy-depletion medium without buffer for 90 min. In the neutral pH condition, cells were incubated in energy-depletion medium with 50 mM imidazole buffer (pH 7.5). In the low-to-neutral pH condition, cells were incubated in energy-depletion medium without buffer for 90 min, followed by an additional 15 min in energy-depletion medium with 50 mM imidazole (pH 7.5). In the recovery experiment, the low-to-neutral pH-treated cells were centrifuged, and then resuspended in SD full glucose medium for 60 min.

When we examined whether raising the pH disassembled the Sup35 condensates in vivo, we first formed condensates in acidic medium, followed by addition of buffer to increase the intracellular pH to neutrality. As shown in Figure 4, after forming the condensates in acidic medium, the condensates persisted when the intracellular pH was increased to neutrality. This was the case even though the Sup35 did not form condensates when yeast were energy-depleted at neutral pH (Figure 4), in agreement with a previous study [3]. Similar results were obtained when the pH was raised to neutrality with imidazole, Tris, or phosphate buffer.

Since Sup45 colocalized with the Sup35 condensates, we expected that Sup45 puncta would have similar properties as the Sup35 condensates. As shown in Figure 4, the effect of pH on the Sup45 puncta was similar to that observed with the Sup35 condensates. The Sup45 did not form puncta when energy-depleted at neutral pH. However, the puncta, which formed when cells were energy-depleted in medium without buffer, persisted when the pH was increased. As expected, both the Sup35 condensates and the Sup45 puncta disassembled when glucose was added. These results show that production of ATP by glycolysis has functions other than just raising the intracellular pH, and it is one or more of these other functions that is necessary for the disassembly of the condensates in vivo. Lowering the pH is sufficient to form the condensates, but raising the pH is only sufficient for disassembly in vitro, but not in vivo.

### 3.4. Properties of the C-Terminal Domain of Sup35

Our results show that the C-terminal domain of Sup35 is essential for Sup35 to form condensates in vivo. Furthermore, like full-length Sup35, C-GFP itself formed bright puncta in energy-depleted cells, but not when the cells were treated with cycloheximide. Therefore, in yeast cells with low intracellular pH, the C-GFP puncta resembled the full-length Sup35 condensates. However, it was previously reported by the Alberti laboratory that the C-terminal domain puncta persisted for a much longer time than the Sup35 condensates when yeast cells with acidified cytosols were resuspended into glucose medium [3]. On this basis, it was proposed that the C-terminal domain puncta were not condensates, but rather were insoluble protein aggregates, just as they observed in vitro with the purified C-terminal domain fragment of Sup35 [3].

**Figure 5:**
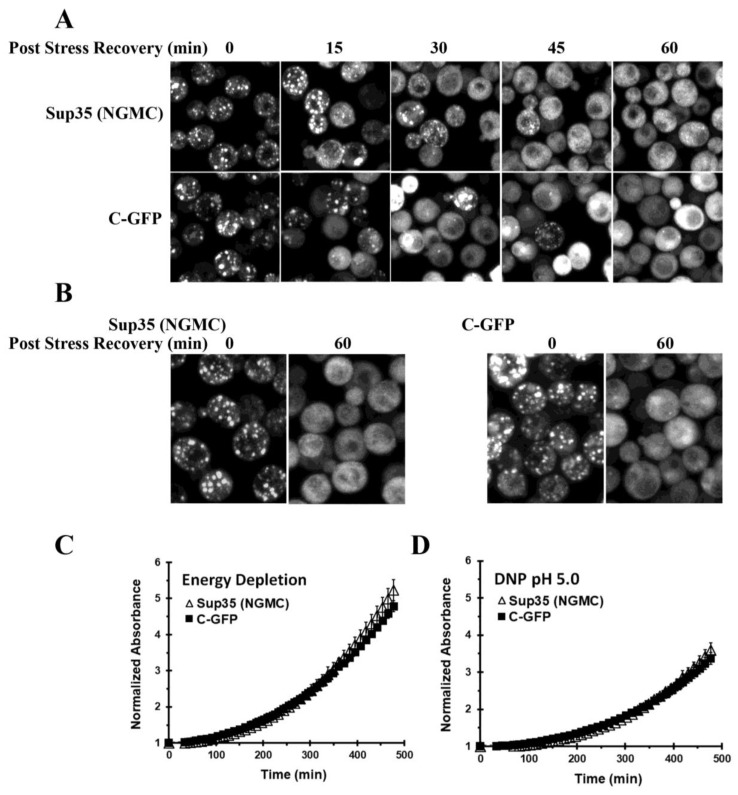
Recovery of yeast expressing either Sup35 (NGMC) or C-GFP following acidification of the yeast cytosol by either energy-depletion or pH stress in SD full medium. (**A**) Maximized Z-stack images of yeast expressing either NGMC (strain 1074) or C-GFP (strain 1618) after energy-depletion for 90 min. The yeast were centrifuged, resuspended in SD medium, and imaged at the indicated times. (**B**) Imaging of yeast expressing either NGMC (strain 1074) or C-GFP (strain 1618) after 90 min in 2 mM DNP and 100 mM phosphate buffer pH 5.0. The yeast were then spun, resuspended in SD medium with glucose for 60 min, and then imaged. (**C**) Time course of growth recovery of yeast following 90 min in an energy-depletion medium. (**D**) Time course of growth recovery of yeast following 90 min in 2mM DNP and 100 mM phosphate buffer pH 5.0. For the time course of recovery in (**C**,**D**), measurements were obtained using three colonies of yeast expressing Sup35 (1074) and three colonies of yeast expressing C-GFP (strain 1618). After energy-depletion or DNP (100 mM phosphate, pH 5.0) treatment, the yeast were spun down and then resuspended in SD full medium. Growth was monitored from the absorbance (OD 600 nm) using a microplate reader. The absorbance measurements from the three colonies were averaged for each condition and then plotted in (**C**,**D**).

To test this proposal, we compared the recovery of yeast expressing either the full-length Sup35 or the C-terminal domain of Sup35. Following energy-depletion of the yeast to reduce the intracellular pH, the yeast were spun down and then resuspended in SD medium with glucose. As shown in Figure 5A, there was not a significant difference in the time course of disassembly of the full-length Sup35 condensates and the C-GFP puncta. Both the GFP-labeled Sup35 and the C-GFP became diffuse in >95% of the cells after 60 min in glucose-containing medium.

To ensure that the method of lowering the pH did not cause misfolding of the C-terminal domain of Sup35, we also lowered the pH using the pH stress method (DNP and phosphate buffer, pH 5.0). Again, we obtained bright C-GFP puncta that disassembled within 60 min in glucose medium (Figure 5B). Therefore, these data show that the C-terminal domain of Sup35 did not form insoluble aggregates when the intracellular pH was acidified.

By comparing the properties of yeast expressing either full-length Sup35 or the C-terminal domain of Sup35, it was reported that the Sup35 condensates conferred a fitness benefit on the yeast [3]. One feature of this fitness benefit was that yeast expressing full-length Sup35 recovered faster from cytosolic acidification when resuspended in glucose medium than yeast expressing only the C-terminal domain of Sup35 [3]. However, since we found that full-length Sup35 and the C-terminal domain of Sup35 disassembled with the same time course, we expected that yeast expressing either full-length Sup35 or the C-terminal domain would show a similar time course of recovery. To compare the time courses of recovery, after cells were energy-depleted, they were spun down, and resuspended in glucose medium. We then monitored the yeast growth by measuring absorbance as a function of time using a microplate reader. As shown in Figure 5C, the growth recovery was not significantly different in yeast expressing full-length Sup35 or yeast expressing only the C-terminal domain of Sup35. We also repeated the experiment on cells that were acidified with 2 mM DNP in phosphate buffer (pH 5.0). Under this condition, we again did not observe a significant difference in the growth recovery between yeast expressing Sup35 and yeast expressing just the C-GFP fragment (Figure 5D). Therefore, there is no evidence that the Sup35 condensates confer this fitness benefit to the yeast.

## 4. Discussion

Recent work has demonstrated that stress induces biophysical changes in the cytosol, including phase separation, which, in turn, produces biomolecular condensates. One of the stresses that causes phase separation and condensate formation is intracellular acidification, which occurs when cells are depleted of energy [3,33,45,46]. Based on the observation that the phase separations of many proteins rely on an intrinsically disordered region that is similar to the prion domain that causes amyloid formation in many yeast proteins, the Alberti laboratory examined the phase separation of the prion-forming protein Sup35 both in vitro using purified Sup35 and in vivo by examining the properties of GFP-labeled Sup35 [3]. The similarity in the properties of the phase-separated liquid droplets of purified Sup35 and the cellular condensates of Sup35 led them to conclude that the prion-domain of Sup35 is driving phase separation in the cell. Therefore, they proposed that the yeast prion proteins can have three different conformations: the properly folded conformation, an amyloid conformation, and the condensate conformation. The condensate conformation of Sup35, which forms at low intracellular pH, disassembles upon addition of glucose, whereas the amyloid conformation, which forms the infectious prion, is stably propagated from mother to daughter cells.

Although the results we obtained using full-length Sup35 in vivo are consistent with previously reported data [3], further characterization of the Sup35 condensates showed that the Sup35 condensates in vivo have very different properties from the Sup35 liquid droplets in vitro. Based on the formation of the Sup35 liquid droplets in vitro, it was proposed that the intrinsically disordered N-terminal prion domain of Sup35 is the driving force for condensate formation in vivo, while the C-terminal domain is unnecessary for condensate formation [3]. In contrast, we found that the C-terminal domain of Sup35 is essential for condensate formation in vivo while the NM domain of Sup35 did not form condensates when the intracellular pH was acidified. Therefore, the intrinsically disordered N-terminal prion domain is not the driving force for Sup35 condensate formation in the cell.

Since the C-terminal of Sup35 forms a complex with Sup45 [5,6,7], this suggested that the role of the C-terminal domain was to bind Sup45 in the condensates. This proposed role is supported by the presence of extensive colocalization of Sup35 condensates with Sup45 puncta when the intracellular pH is acidified. As for the driving force for condensate formation, our data indicate that this is mRNA, which is released from the ribosomes when cells are energy-depleted [47]. We found that the Sup35, Sup45, and the C-terminal domain of Sup35 did not form bright puncta when cells were treated with cycloheximide to prevent mRNA release from the polysomes [29,30,31,32,33]. However, mRNA is not sufficient to cause condensate formation of Sup35 since energy depletion at neutral pH did not produce condensates. This shows that both low pH and mRNA are necessary for Sup35 condensate formation.

Not surprisingly, since our results show that mRNA is critical for condensate formation, which acts as a scaffold to recruit many different ribonucleoproteins, we found that when cells are energy-depleted, both Sup35 and Sup45 colocalize with Pub1, a stress granule marker protein [16]. We also found that the C-terminal domain of Sup35 colocalized with Pub1, but to a lesser extent than full-length Sup35. The fact that the C-terminal domain of Sup35 and the full-length Sup35 differ in this respect show that the NM domains are altering the behavior of the C-terminal domain when Sup35 forms condensates. This effect of NM on complex assembly may account for the observation that the M-domain of Sup35 was previously reported to affect the pH dependence of condensate formation [3]. When the cluster of negative charges in the M-domain was mutated, the mutated Sup35 formed condensates at a higher pH in energy-depleted cells than wild-type Sup35.

We also found that Sup35 condensates that formed in vivo did not disassemble when pH was increased, unlike the phase-separated Sup35 liquid droplets formed in vitro [3]. Disassembly did occur when glucose was added to the medium. Under this condition, the yeast produces ATP, which suggests that ATP is playing another role in condensate disassembly in addition to increasing the intracellular pH of the yeast. It was previously shown that the ATP-binding chaperone Hsp104 is not necessary for disassembly of the Sup35 condensates [48], but other ATP-binding chaperones, e.g., members of the Hsp70 family, may be required for condensate disassembly. Another possibility is that ATP is required for the phosphorylation of certain key proteins. For example, the Ross laboratory found that Sky1 kinase regulates stress granule dissolution by phosphorylating NPL2, an RNA-shuttling protein [49].

It was previously proposed that formation of Sup35 condensates, but not the formation of the C-terminal domain puncta, enhances yeast survival under stress conditions [3,48]. This proposal was based on their observation that the C-terminal domain formed puncta similar to the Sup35 condensates when the yeast cells were acidified, but unlike the Sup35 condensates, the C-terminal puncta did not disassemble upon addition of glucose. This led them to propose that the C-terminal domain formed insoluble misfolded aggregates at low pH. In contrast to this result, we observed that, following a decrease in pH due to either DNP/phosphate treatment or energy-depletion medium, both the Sup35 condensates and the C-terminal domain puncta disassembled within an hour at about the same rate. Consistent with the C-terminal domain not being misfolded following acidification, yeast expressing Sup35 or the C-terminal domain both showed a similar time course of growth recovery following energy-depletion or pH stress with DNP when incubated in glucose-containing medium. Therefore, there is no evidence that expression of Sup35, per se, confers a fitness benefit on the yeast; acidification itself confers a fitness benefit by causing many proteins to form condensates [45,46,50].

In conclusion, we found that the properties of the Sup35 biomolecular condensates formed in vivo are very different than those reported for the Sup35 liquid droplet formed in vitro. This highlights the difficulty in applying the biophysical characterization of proteins in vitro to what occurs in the in vivo cellular environment [51]—the cell contains many more proteins and other molecules, e.g., RNA, that can affect the phase separation process. To overcome this limitation, instead of studying the liquid phase separation of just one protein, the Parker and Rosen laboratories were able to reconstitute P-bodies in vitro that had similar properties as cellular P-bodies by combining seven proteins that are highly concentrated in P-bodies with RNA [38,42]. Similarly, to get at a true understanding of Sup35 condensate formation in vivo, a biochemical characterization of the condensate formation in vitro will no doubt require purification not only of Sup35, but, at a minimum, Sup45 and mRNA as well.

## Figures and Tables

**Table 1:** Yeast strains.

Name	Genotype
1074	*MAT*a, *kar1-1, SUQ5, ade2-1, his3Δ202, leu2Δ1, trp1Δ63, ura3-52, sup35::NGMC*
779-6A	*MAT*a*, kar1-1, SUQ5, ade2-1, his3Δ202, leu2Δ1, trp1Δ63, ura3-52*
1618 C-GFP	780-1D, *sup35::KanMX/pAG415-PSUP35-SUP35-C-GFP*
1905	*MAT*a*, kar1-1, SUQ5, ade2-1, leu2Δ1, trp1Δ63, ura3-52, his3::SUP45-GFP-HISMX6*
780-1D	*MAT*a *kar1*-*1 SUQ5 ade2*-*1 his3*Δ*202 leu2*Δ*1 trp1*Δ*63 ura3*-*52 sup35*::*KanMX*/pJ533 (*URA3, SUP35*)
74D-694	*MAT*a: *ade1-14*, *trp1-289*, *his3*Δ*-200*, *ura3-52*, *leu2-3*, *112*
L2888	*MAT*alpha*: ade1-14*, *trp1-289*, *his3*Δ*-200*, *ura3-52*, *leu2-3*, *112, sup35::NGMC*

**Table 2:** Plasmids.

Name	Description	Reference
TH1123	P*_SUP35_*-*SUP35-C-GFP*., CEN, *LEU2*	[3]
pXZ122	P*_SUP35_*-*SUP35-NM-GFP*., CEN, *LEU2*	This study
pXZ510	P*_SUP35_*-*SUP35-NRMC*, CEN, *LEU2*	This study
pRP1661	P_PUB1_-*PUB1-mCherry*, CEN, *URA3*	[16]
115697	p426Met25-sfpHluorin (MRV55)	[34]

**Table 3:** Intracellular pH of yeast.

Conditions	pH
SD Full	7.4
10 min ED	5.9
20 min ED	5.4
30 min ED	5.4
2 h ED	5.4
2 h ED + 50 mM Tris (pH 7.8)	7.5
2 h ED + 50 mM imidazole (pH 7.5)	7.5
2 h ED + 50 mM phosphate (pH 7.5)	6.8
2 h ED + 30 min 50 mM Tris (pH 7.5)	7.5
2 h ED + 30 min 50 mM imidazole (pH 7.5)	7.4
2 h ED + 30 min 50 mM phosphate (pH 7.8)	6.8

## Data Availability

The data supporting the findings of this study are available on the following website: https://doi.org/10.25444/nhlbi.23818215 (accessed on 14 August 2023).

## Supplementary Materials

The following supporting information can be downloaded at: https://www.mdpi.com/article/10.3390/biom13091370/s1, Figure S1: The NM domains of Sup35 do not form condensates in yeast under different conditions; Figure S2: Stress granules form faster than Sup35 condensates in energy-depleted yeast; Table S1: Imaris parameters for finding colocalization of aggregates.
