# Supplementary material for: The Properties and Domain Requirements for Phase Separation of the Sup35 Prion Protein In Vivo
Source: Biomolecules. 2023 Sep 10;13(9):1370. doi: 10.3390/biom13091370 (PMC10526957; doi:10.3390/biom13091370)
Supplement: Supplementary file 1 [file biomolecules-13-01370-s001.zip › biomolecules-2564608-supplementary.pdf]

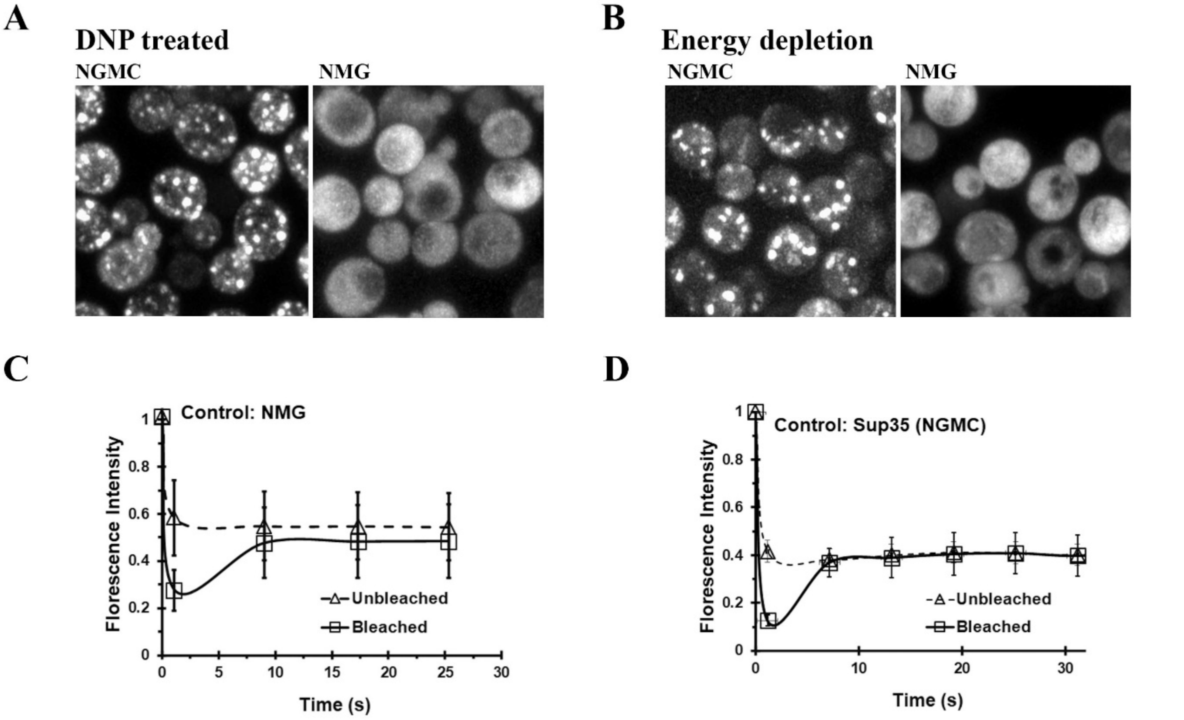

Figure S1. The NM domains of Sup35 do not form condensates when yeast are treated with 2,4 DNP or energy depleted in a different yeast strain. A, Yeast expressing either Sup35(NGMC) or NM-GFP in the 779-6A strain were incubated in 2 mM DNP, 100 mM phosphate buffer (pH 5.0) for 60 min prior to imaging. B, Yeast expressing either Sup35(NGMC) or NMG in the 74D-694 strain were incubated in SD media without glucose with 20 mM deoxyglucose and 10 μM Antimycin A for 60 min prior to imaging. C, Fluorescence recovery after photobleaching of yeast expressing NM-GFP in SD media with glucose. D, Fluorescence recovery after photobleaching of yeast expressing NGMC in SD media with glucose. The yeast expressing NGMC in the 779-6A and yeast expressing NGMC in the 74D-694 strain are listed in Table 1 as 1074 and L2888 strains, respectively.

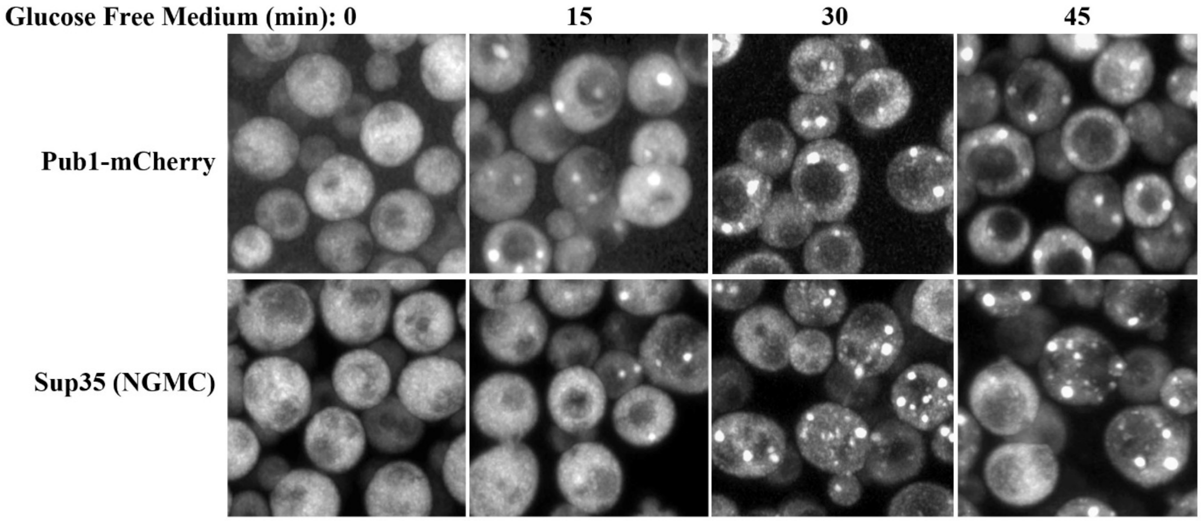

Figure S2. Stress granules form at a faster rate in glucose free media than Sup35 condensates. Maximized Z-stack confocal images of yeast either expressing Pub1-mCherry, a stress granule marker protein, or Sup35 (NGMC) when incubated for the indicated times in glucose free media. The 779-6A strain was transformed with Pub1-mCherry plasmid, whereas the NGMC was expressed from the *SUP35* locus in the 779-6A strain (referred to as the 1074 strain in Table1).

Table S1: Imaris parameters for finding aggregates using spot detection.*

[Algorithm]
Enable Region Of Interest = false
Enable Region Growing = true
Enable Tracking = false
[Source Channel]
Source Channel Index = 1
Estimated XY Diameter = 0.426 um
Estimated Z Diameter = 0.852 um
Background Subtraction = true
[Classify Spots]
"Quality" above automatic threshold
[Spot Region Type]
Region Growing Type = Local Contrast
[Spot Regions]
Region Growing Automatic Threshold = true
Region Growing Manual Threshold = 10.498
Region Growing Diameter = Diameter From Border
Create Region Channel = false

* To determine colocalization, the Spots Colocalization Tool was used with a parameter of 0.25 (empirically determined).
